# Diving Responses in Experienced Rebreather Divers: Short-Term Heart Rate Variability in Cold Water Diving

**DOI:** 10.3389/fphys.2021.649319

**Published:** 2021-04-07

**Authors:** Richard V. Lundell, Laura Tuominen, Tommi Ojanen, Kai Parkkola, Anne Räisänen-Sokolowski

**Affiliations:** ^1^Diving Medical Centre, Finnish Defence Forces, Helsinki, Finland; ^2^Department of Anaestesia, Tampere University Hospital, Tampere, Finland; ^3^Finnish Defence Research Agency, Finnish Defence Forces, Tuusula, Finland; ^4^Faculty of Medicine and Health Technology, Tampere University, Tampere, Finland; ^5^Department of Leadership and Military Pedagogy, National Defence University, Helsinki, Finland; ^6^Department of Pathology, Helsinki University Hospital, and University of Helsinki, Helsinki, Finland

**Keywords:** HRV, cold diving, Arctic diving, diving response, rebreather diving, diving reflex, technical diving

## Abstract

**Introduction:**

Technical diving is very popular in Finland throughout the year despite diving conditions being challenging, especially due to arctic water and poor visibility. Cold water, immersion, submersion, hyperoxia, as well as psychological and physiological stress, all have an effect on the autonomic nervous system (ANS).

**Materials and methods:**

To evaluate divers’ ANS responses, short-term (5 min) heart rate variability (HRV) during dives in 2–4°C water was measured. HRV resting values were evaluated from separate measurements before and after the dives. Twenty-six experienced closed circuit rebreather (CCR) divers performed an identical 45-meter decompression dive with a non-physical task requiring concentration at the bottom depth.

**Results:**

Activity of the ANS branches was evaluated with the parasympathetic (PNS) and sympathetic (SNS) indexes of the Kubios HRV Standard program. Compared to resting values, PNS activity decreased significantly on immersion with face out of water. From immersion, it increased significantly with facial immersion, just before decompression and just before surfacing. Compared to resting values, SNS activity increased significantly on immersion with face out of water. Face in water and submersion measures did not differ from the immersion measure. After these measurements, SNS activity decreased significantly over time.

**Conclusion:**

Our study indicates that the trigeminocardiac part of the diving reflex causes the strong initial PNS activation at the beginning of the dive but the reaction seems to decrease quickly. After this initial activation, cold seemed to be the most prominent promoter of PNS activity – not pressure. Also, our study showed a concurrent increase in both SNS and PNS branches, which has been associated with an elevated risk for arrhythmia. Therefore, we recommend a short adaptation phase at the beginning of cold-water diving before physical activity.

## Introduction

Recreational diving is the most common form of diving. Sometimes also referred to as non-technical diving, this form of diving takes place in open water, compressed air is used as breathing gas and no decompression procedures are required while surfacing. If dives are intended to be deeper and last longer, more advanced dive planning is required. This includes the usage of special breathing gas mixtures, more technical equipment and a planned decompression procedure. This form of diving is referred to as technical diving (Doolette and Mitchell, 2013; Mitchell and Doolette, 2013). During the new millennium, technical diving has been increasing in popularity. Technical diving, and especially the subcategory of closed circuit rebreather (CCR) diving, has caused a shift toward a more demanding type of diving. Technical diving with CCR units bring advantages compared to open circuit scuba, for example lower gas consumption and warmer and more humidified breathing gas (Mitchell and Doolette, 2013). In Finland, diving conditions are challenging – water temperature throughout the year is 4°C in depths below 30 meters and during the winter, surface water temperatures vary from −1 to 2°C. Despite the freezing cold water, Finnish technical divers dive throughout the year (Lundell et al., 2019). Moreover, in Finland, poor visibility in most diving locations, especially in seas and lakes, brings additional challenges to the already demanding conditions. For these reasons, technical divers tend to dive in old water-filled mines that have clear water and better visibility.

When diving, the body reacts to cold through autonomic nervous system (ANS) mediated thermoregulatory mechanisms. A decrease in core temperature causes e.g., vasoconstriction, shivering and muscle stiffness (Wittmers, 2001). Immersion in cold water stimulates both the parasympathetic (PNS) and sympathetic (SNS) branches of the ANS (Doubt, 1996; Mourot et al., 2007). Submersion causes physiological and psychological stress which activates the SNS (Doubt, 1996; Schirato et al., 2018, 2020). The human diving responses mainly cause an increase in PNS activity and can be divided into separate physiological responses to immersion and submersion. Hydrostatic pressure causes many physiological changes, such as an increase in thoracic blood volume, which alters PNS activity and leads to bradycardia and an increase in stroke volume and cardiac output (Epstein, 1992; Sramek et al., 2000; Florian et al., 2013). Face immersion stimulates the trigeminocardiac reflex also characterized by bradycardia due to vagal activity (Gooden, 1994). Hyperoxia also causes an increase in PNS activity (Lund et al., 2000, 2003; Barbosa et al., 2010) and attenuates SNS activity (Yamauchi et al., 2002).

Heart rate variability (HRV) can be used to indirectly study changes in activity of the ANS branches. Short-term HRV is influenced by many factors, including dynamic PNS/SNS balance, as well as respiration via the respiratory sinus arrhythmia, heart and vascular tone via baroreceptor and cardiac stretch receptor activity, the central nervous system (CNS), the endocrine system and chemoreceptor activity (Karemaker, 2009; Gevirtz et al., 2016; Shaffer and Ginsberg, 2017). Diving predominantly causes an increase in PNS activity. Studies have been performed in hyperbaric chambers (Lund et al., 2000, 2003) and in pool-like conditions (Schipke and Pelzer, 2001; Chouchou et al., 2009), with various dives in different depths (Noh et al., 2018), but to the best of our knowledge, only one involved cold water conditions (Lundell et al., 2020). In the aforementioned literature, prominent PNS activation occurred, whereas SNS activity decreased. Only one study presented a decrease in PNS measures in a diver that performed a psychologically challenging dive in shallow, warm water (Flouris and Scott, 2009).

The aim of this study was to examine ANS responses in experienced CCR divers with short-term HRV measures from decompression dives to 45 meters in arctic temperatures. Special focus was on initial ANS changes – the “diving responses” at the beginning of the dives.

## Materials and Methods

### Subjects

Twenty-six experienced, healthy subjects, male (*n* = 23) and female (*n* = 3), took part in the tests. The subjects were recruited from the Finnish recreational technical diving community (*n* = 23) and Finnish Defense Forces (*n* = 3). Each diver performed one dive. All subjects participated voluntarily and gave their informed consent for the study. Divers did not receive any financial gain for participation in the research dives. Each subject filled out a health survey, and a physician performed a fit-to-dive examination on the morning of the dive. Only healthy subjects were allowed to dive. All divers had insurance covering potential adverse effects of the dives.

The study adhered with the Declaration of Helsinki. Ethical approval was granted by the Ethical Committee of Helsinki University Hospital (HUS/976/2019). Research permission was received from both Helsinki University Hospital (HUS/44/2019) and the Finnish Defense Forces Logistics Command (AP22409, 18.12.2019).

### Diving Equipment

Divers used their own diving equipment during the tests. These included their usual undergarments and dry suits. Divers were allowed to wear heating vests under their dry suits but they were instructed not to use them, except in case of emergency or strong discomfort due to cold. All subjects used their own CCR unit [JJ-CCR (*n* = 17), Megalodon (*n* = 1), rEVO (*n* = 4), AP Inspiration evolution (*n* = 2), Sentinel (*n* = 1), T-Reb (*n* = 1)].

### Preparation and Diving Procedure

No alcohol was allowed for 24 h before the dive. During the diving day, subjects were instructed to hydrate according to their regular routines until 2 h before the dive. Thereafter, 5 dl of sports drink (Gatorade, PepsiCo, Nordic Finland Ltd., Helsinki, Finland) was consumed.

The test dives occurred on two weekends in January at the old water-filled mine in Ojamo (Lohja, Finland). Diving conditions were normal for this time of year: the water was covered with a thin sheet of ice, water temperature was 2°C near the surface and 4°C at a depth of 45 meters, and visibility was approximately 10 meters. To ensure identical dives for all subjects, a pre-set line from surface to bottom was set.

Preparations for dives were made in a room with constant air temperature (19°C). Bodyguard two HRV devices (Firstbeat Technologies Ltd., Jyväskylä, Finland) were attached and four skin temperature electrodes were placed at standard measuring spots according to the International Organization for Standardization’s standard (neck, scapula, hand and shin) [ISO 15027-3:2012(E)].

Just before the dives, all subjects were instructed to follow a strict protocol for the start of the dive: (1) immersion with face out of water (5 min), (2) face in water at surface (5 min) (face was in water practically all time), (3) start of dive and submersion to a depth of six meters (5 min). All divers followed the protocol successfully. After this, the divers followed a pre-set line down the slope to the depth of 45 meters. Descending to a depth of 45 meters took approximately 5 min and the divers spent approximately 18 min at the bottom.

**FIGURE 1:**
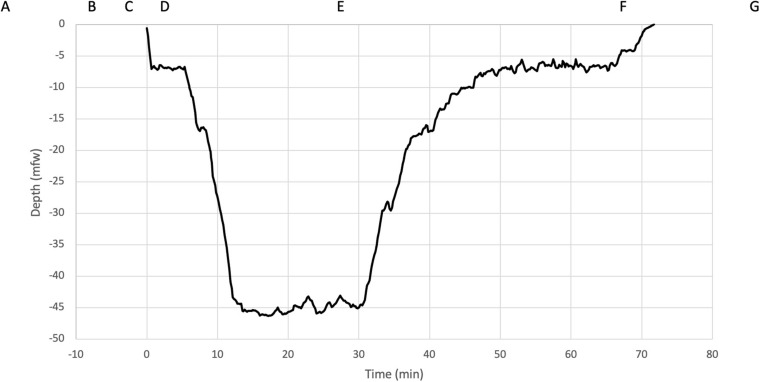
Five-minute HRV measurements were performed at the following time points indicated in boxes: **(A)** rest before the dive, **(B)** immersion, **(C)** face in the water, **(D)** submersion, **(E)** bottom, just before starting ascent, **(F)** just before surfacing and **(G)** rest after the dive. The dive profile (diver number nine) was downloaded from the dive computer and is shown with corresponding depths.

During the ascent, divers followed an earlier defined decompression profile: Suunto Fused^TM^ RGBM 2 (Suunto Ltd., Vantaa, Finland) with personal adjustment +2 (Suunto EON Core and Suunto D5 dive computers). All CCR devices used standardized diluent trimix 20/40 and the oxygen controllers maintained constant oxygen partial pressure in breathing loop (pO_2_ 70 kPa in the beginning of dive and pO_2_ 120 kPa at the bottom depth and during ascent). With this set up, the total dive time was approximately 66 min. One of the divers (diver number nine) dive profile is presented in Figure 1.

### Measurements

The HRV Bodyguard two device (Firstbeat Technologies Ltd., Jyväskylä, Finland) was used to record R to R wave measures at a 1000 Hz sampling frequency. Short-term (5 min) HRV was analyzed with the Kubios HRV Standard program (version 3.3.1, Kubios Ltd., Kuopio, Finland) for time points presented in Figure 1. The resting values were recorded in a supine position in thermoneutral conditions before (39–79 min before start of dive) and after the dives (59–114 min after surfacing). The automatic artifact correction function of the program was used to correct corruption in data and the program’s time series trend removal tool was used for each subject before analysis (Tarvainen et al., 2014).

Activity of the ANS branches was evaluated with the help of the program’s PNS and SNS indexes, which provide reliable estimates compared to normal resting values (Sahoo et al., 2019). These indexes are based on known HRV parameters that reflect PNS and SNS activity (Tarvainen et al., 2014). Moreover, we used three time domain and five frequency domain measures. Time domain measures were: mean heart rate (HR_*mean*_) (bpm), standard deviation of NN intervals (SDNN) (ms) and root mean square of successive RR interval differences (RMSSD) (ms). Frequency domain measures were: absolute total power (TP) (ms^2^), absolute power of the very low frequency band (VLF) (ms^2^), absolute power of the low frequency band (LF) (ms^2^), absolute power of the high frequency band (HF) (ms^2^) and ratio of LF to HF power (LF/HF) (%).

Area weighted skin temperature (Tskin) was calculated from the skin temperature measures with the ISO Standard weighting coefficients at 5-min intervals (Tskin = neck^∗^0.28 + scapula^∗^0.28 + hand^∗^0.16 + shin^∗^0.28) (The International Organization for Standardization, 2012). Subjective evaluation of thermal comfort with a scale of 1–10 (1 = freezing cold, 5 = thermoneutral, and 10 = burning hot) was done at 10-min intervals (at run time 30, 40, 50, and 60 min).

### Statistics

HRV variables are presented using medians and interquartile ranges (IQR). Comparisons were made using Mann–Whitney *U* tests for the changes of the variables from pre-dive rest to immersion and post-dive rest, whereas immersion was compared against the rest of the time points.

The relation between the temperate measurements and diving time was investigated using linear mixed models with both random intercepts and slopes for each diver. The p-values were calculated using Satterthwaite’s estimation for the degrees of freedom.

Significance level was set to 0.05. All analyses were done using R version 4.0.3 (R Core Team, 2020) and visualization was done using the ggplot2 package (Wickham, 2016). The linear mixed models were fitted using the lme4 package (Bates et al., 2015).

## Results

### Description of Study Population

The study dive was performed in accordance with the certification and experience level of the divers. All divers were trained for overhead environment diving and the test dive was performed at a dive site already familiar to them. Twenty-six CCR divers (3 female, 23 male) completed a decompression dive as planned. Subject demographics, measured with InBody 720 composition analyzer (Biospace Ltd., Seoul, South Korea), are shown in Table 1. Four of 26 divers had a pre-existing medical condition, i.e., controlled hypertension, but only two had medication for hypertension (olmesartan and losartan).

**TABLE 1:** Demographics of 26 study subjects.

Demographics of 26 study subjects	Range	Mean
Age (years)	28–57	44.0
Height (m)	1.63–1.89	1.79
Weight (kg)	65.4–140.5	88.4
BMI (kg/m^2^)	23.4–39.8	27.4
Body muscle mass (kg)	28–51.5	38.9
Body fat mass (kg)	4.8–65.8	20.0
Fat (%)	6.2–46.8	21.7
Systolic blood pressure (mmHg)	125–210	146
Diastolic blood pressure (mmHg)	73–122	92
Diving experience (years)	6.1–30	13.9
Dives (*n*)	300–1950	845

*Body composition and blood pressure were measured before the dives. All divers were “very experienced” but exact information on diving years and number of dives was missing for three divers. Hence, this information is based on 23 divers in the study. Mean and range shown.*

### HRV Data

**FIGURE 2:**
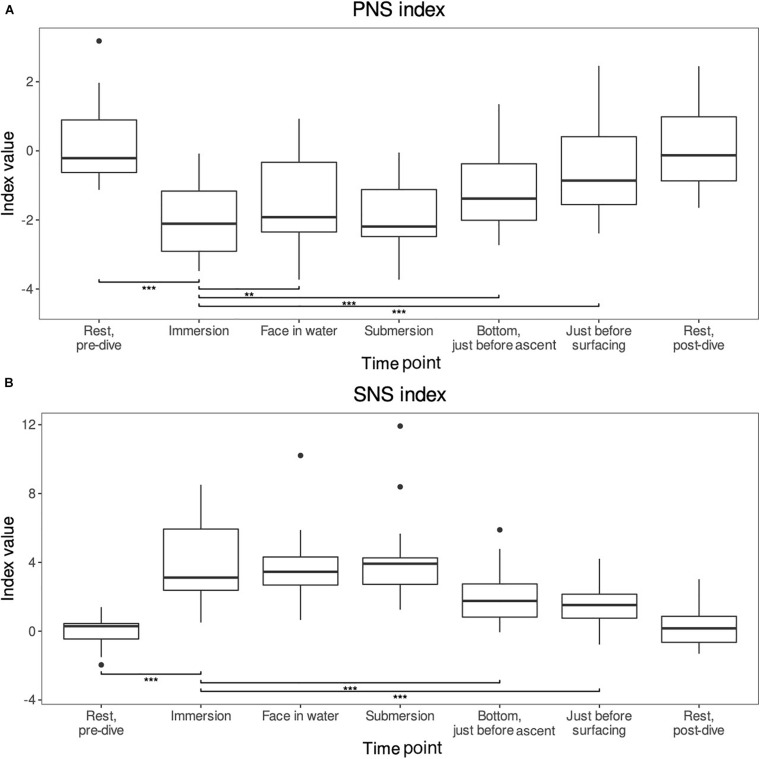
**(A,B)** The PNS and SNS indexes reported by the Kubios HRV standard program. These indexes provide reliable estimates of the autonomic nervous system activities compared to normal resting values (Sahoo et al., 2019). For comparisons, the level of significance is reported **p < 0.01 and ***p < 0.001. Means and range shown (*n* = 26).

The changes and test results for both of the indexes are shown in Figures 2A,B.

The PNS index was around zero [−0.21 (range −0.63–0.90)] during the pre-dive rest period and varied by −2.20 (−2.70–1.65) on immersion (*p*-value < 0.001). From immersion it increased significantly when the face was immersed in water. Also, an increase was seen just before the decompression and just before surfacing, the changes were 0.54 (−0.04–1.07) *p* < 0.01, 0.84 (0.35–1.39) *p* < 0.001, and 1.67 (0.71–2.40) *p* < 0.001, respectively. Though submersion did not differ statistically significantly from the immersion, the general trend of the index was a decrease during immersion and then slow increase back to pre-dive rest levels which were reached during the post-dive rest period (difference *p*-value = NS).

The SNS index was also around zero [0.29 (−0.46-0.45)] during the pre-dive resting phase. The index increased by 3.77 (2.53–5.44) *p* < 0.001 on immersion. After immersion, the index dropped statistically significantly when the diver reached the bottom [−1.69 (−2.79–0.99), *p* < 0.001] and just before surfacing [−2.41 (−4.25–1.25), *p* < 0.001]. The face in water and submersion periods did not differ from the immersion period (*p* = NS). The general trend was a mirror image of the PNS index: sharp increase during immersion and then slow decline back to the pre-dive rest levels which were once again reached during the post-dive rest period (pre- vs post-dive rest period *p*-value = NS).

**FIGURE 3:**
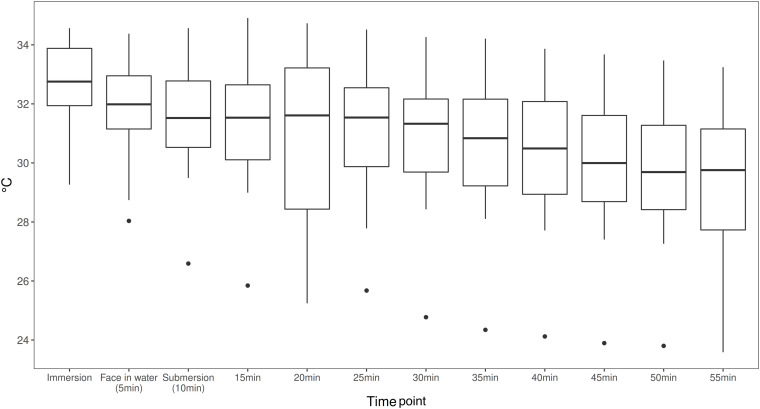
Area weighted skin temperature (Tskin) calculated from the four skin temperature measures with standardized weighting coefficients at a 5-min interval during the dives (Tskin = neck*0.28 + scapula*0.28 + hand*0.16 + shin*0.28) [ISO 15027-3:2012(E)]. Means and range shown (*n* = 26).

The linear mixed model estimates a temperature drop of Tskin per minute of −0.048°C with a SE of 0.005 and *p*-value < 0.001. On average, divers start with a temperature of around 32.26°C, which then dropped by 2.68°C during the 55-min dive. Values for Tskin are presented in Figure 3. Subjective evaluation of thermal comfort is presented in Table 2.

**TABLE 2:** Subjective evaluation of thermal comfort on a scale of 1–10 (1 = freezing cold, 5 = thermoneutral and 10 = burning hot) was done at 10-min intervals (at run time 30, 40, 50, and 60 min).

Thermal comfort	30 min	40 min	50 min	60 min
Runtime	Range (mean)	Range (mean)	Range (mean)	Range (mean)
1 = freezing, 5 = normal	3–5 (4.3)	3–5 (3.8)	2–5 (3.4)	2–4 (3.4)

*Means and (range) shown (*n* = 26).*

Results for other HRV parameters are presented in the Supplementary Figure 1A–H and Supplementary Table 1.

## Discussion

To our knowledge, this is the first HRV study on rebreather divers. As shown in earlier studies, an increased oxygen partial pressure activates the PNS branch of the ANS (Lund et al., 2000). All divers in our study used the same oxygen partial pressure. This is significantly higher than oxygen partial pressure at atmospheric ambient pressure (70–120 kPa vs. 21 kPa). Therefore, the breathing gas most likely contributed to the increase in PNS activity during the dives.

As PNS activity still increased significantly from higher ambient pressure (measured before decompression) to lower ambient pressure (last measured just before surfacing), it is unlikely that the breathing gas would have had the biggest role in contributing to PNS activation.

In an earlier study, we discussed the role of different parts of the diving reflex in contributing to PNS activation at the beginning of diving in very cold conditions (Lundell et al., 2020). Because divers submerged directly when entering the water in that study, we could only speculate on the roles of the following parameters. Firstly, pressure induces centralization of peripheral blood volume due to hydrostatic pressure, causing activation of cardiac stretch receptors and baroreceptors (Chouchou et al., 2020). Secondly, the role of the trigeminocardiac reflex (Lemaitre et al., 2015) and thirdly, the role of cold induces PNS activation [mainly the same mechanism as in pressure (Ihsan et al., 2016)]. Due to the study setting in this research, we could view the aforementioned parameters separately. This showed that pressure solely due to immersion with face out of water did not seem to influence PNS activity. The trigeminocardiac reaction caused a strong PNS activation, seen when divers put their face in the water, but for the next measure PNS activity decreased again, indicating that this reaction is short-lived.

These results give support to the hypothesis that was presented in our previous work: the trigeminocardiac part of the diving reflex causes the initial strong PNS stimulus, but its effect also diminishes quickly. The secondary PNS activation is most likely caused by cold, and pressure induces changes in blood distribution as discussed in our previous study (Lundell et al., 2020). As seen in this study, temperature, estimated from Tskin, decreased during the dives significantly. This finding is supported by the subjective reports of cold sensation. Based on the last measurement before surfacing, pressure surprisingly did not seem to have a larger impact on PNS activity. This would indicate that cold is actually the greatest promoter of PNS over time. However, this finding requires further investigation.

In this study, the SNS increase at the beginning of the dives was most likely due to a reaction to cold. A similar finding was previously described by Youssef et al. (2018). The increase in SNS activity up until the submersion measurement was a somewhat surprising finding. Nonetheless, it could be explained with the fact that divers had to swim forward to reach a spot at six meters of depth where they stayed for a 5-min period. This physical activity, although not strenuous, could have caused the SNS activation seen in the submersion measure. From this measure forward, SNS activity decreased over time, as in our earlier study (Lundell et al., 2020).

Compared to earlier literature, this study had the largest group of divers all performing a similar dive. The results were also in line with earlier HRV studies for diving (Schipke and Pelzer, 2001; Zenske et al., 2020). However, it also provided new findings and confirmed theories discussed in earlier research.

Our study has some limitations. It was not done in controlled laboratory-like conditions, such as a wet chamber, but in usual dives with limited physical activity. This having been said, the water temperature was constant during all dives and divers were instructed not to move too promptly during the dives. Nevertheless, subjects had to swim forth for a while during descent to the correct destination at the bottom of the mine, possibly influencing ANS functions. In addition, the HRV rest measurements before and after the dives were performed at a thermoneutral temperature, while measurements from the dives were performed with diving undergarments on. Thus, the measurement conditions were not fully comparable. Still these measurements were more controlled than in our earlier HRV study on cold-water divers.

## Conclusion

Our study indicates that the trigeminocardiac part of the diving reflex causes the first PNS increase at the beginning of the dive. This reaction seems to decrease quickly. Our study also showed a concurrent increase in both the SNS and PNS branches of the ANS, which has been associated with an elevated risk for arrhythmia.

For this reason, we recommend a short adaptation phase at the beginning of cold-water diving before commencing physical activity. The ideal length of the adaptation phase cannot be determined by the results of our study. However, we suggest that it should span the trigeminocardiac part of the diving reflex, which according to our results lasts under 5 min.

After the first trigeminocardiac activation of the PNS, cold surprisingly seemed to be the most prominent promoter of PNS activity – not pressure – according to this study.

## Data Availability Statement

The raw data supporting the conclusions of this article will be made available by the authors, without undue reservation.

## Ethics Statement

The studies involving human participants were reviewed and approved by Ethical Committee of Helsinki University Hospital (HUS/976/2019). The patients/participants provided their written informed consent to participate in this study.

## Author Contributions

RL, LT, KP, and AR-S planned the study and consulted TO during the planning phase. All authors participated in data gathering and processing. RL and LT were the main writers of the manuscript and participated in data analysis. KP, TO, and AR-S participated in the writing of the manuscript.

## Conflict of Interest

The authors declare that the research was conducted in the absence of any commercial or financial relationships that could be construed as a potential conflict of interest.

## Supplementary Material

Supplementary Figure 1 **(A–H)** Eight 5-min HRV measures: three time domain measures [**(A)** mean heart rate (HRmean), **(B)** standard deviation of NN intervals (SDNN), and c. root mean square of successive RR interval differences (RMSSD)], and five frequency domain measures [**(D)** absolute power of the very low frequency band (VLF power), **(E)** absolute power of the low frequency band (LF), **(F)** absolute power of the high frequency band (HF), **(G)** absolute total power (TP), and **(H)** ratio of LF to HF power (LF/HF)]. The level of significance is reported *: *p* < 0.05, **: *p* < 0.01, and ***: *p* < 0.001. Means and range shown (*n* = 26).

Supplementary Table 1 Eight 5-min HRV measures: three time domain measures [mean heart rate (HRmean), standard deviation of NN intervals (SDNN), and root mean square of successive RR interval differences (RMSSD)], and five frequency domain measures [absolute power of the very low frequency band (VLF power), Absolute power of the low frequency band (LF), absolute power of the high frequency band (HF), absolute total power (TP), and ratio of LF to HF power (LF/HF)]. Pre-dive rest values were compared to immersion values and to post-dive rest values. Moreover, comparison was made between immersion values and the other four in-water measures (face-in-water, submersion, bottom and just before surfacing). Mean and (range) shown (*n* = 26).
