# Supplementary figures and images for: Diving Responses in Experienced Rebreather Divers: Short-Term Heart Rate Variability in Cold Water Diving
Source: Front Physiol. 2021 Apr 7;12:649319. doi: 10.3389/fphys.2021.649319 (PMC8058382; doi:10.3389/fphys.2021.649319)

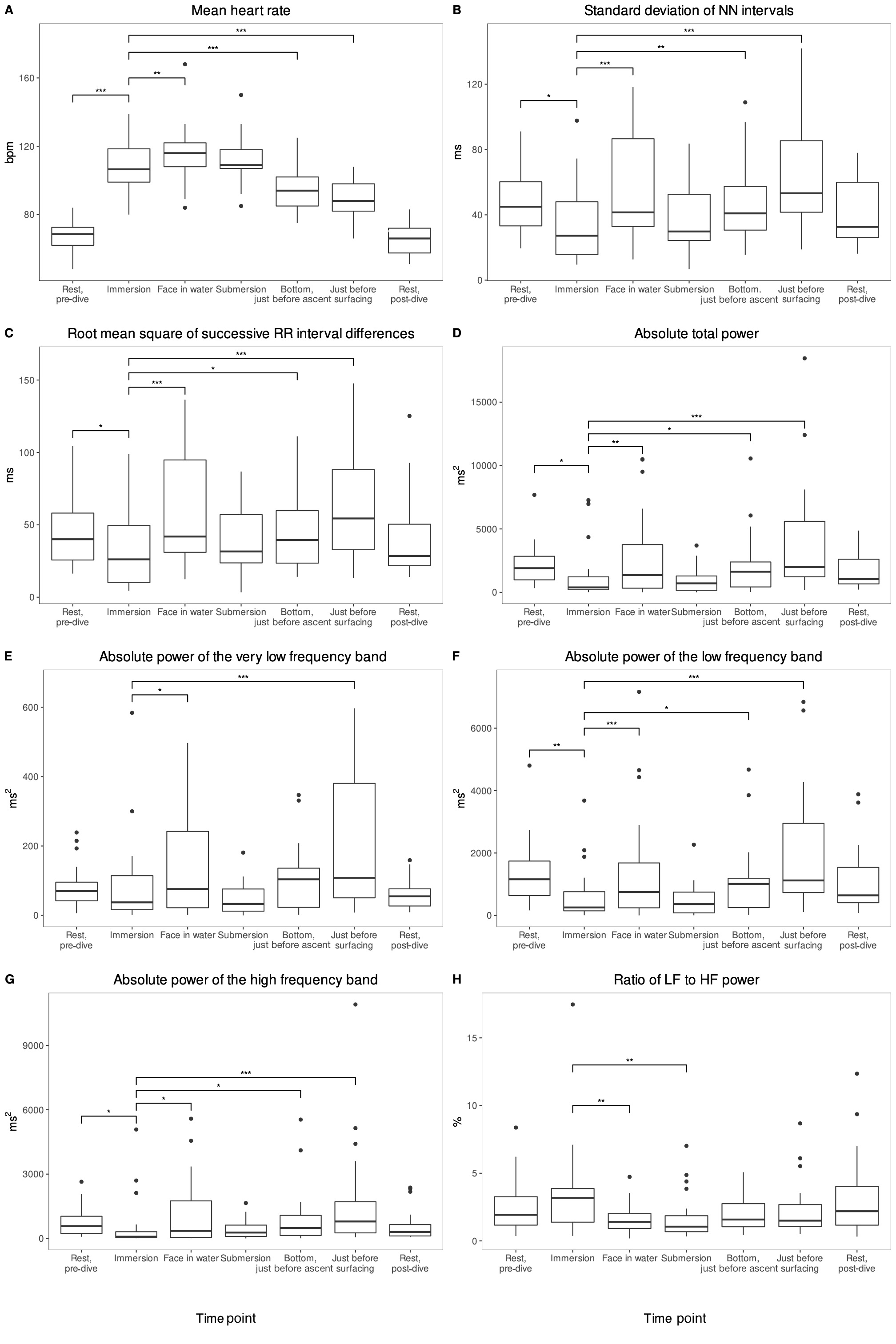

Supplement: Supplementary Figure 1 — (A–H) Eight 5-min HRV measures: three time domain measures [(A) mean heart rate (HRmean), (B) standard deviation of NN intervals (SDNN), and c. root mean square of successive RR interval differences (RMSSD)], and five frequency domain measures [(D) absolute power of the very low frequency band (VLF power), (E) absolute power of the low frequency band (LF), (F) absolute power of the high frequency band (HF), (G) absolute total power (TP), and (H) ratio of LF to HF power (LF/HF)]. The level of significance is reported *: p < 0.05, **: p < 0.01, and ***: p < 0.001. Means and range shown (n = 26). [file Image_1.JPEG]
